# Oxygen at Oxford

**Published:** 1892-04-02

**Authors:** 


### The Doctor's Armchair.

### OXYGEN AT OXFORD.

Oxygen in certain parts of Oxford seems to be like the snakes in Iceland—it does not exist. It is an easy step from Oxford to Mr. Matthew Arnold and "Philistinism." Arnold loved Oxford, and much as he regretted her "inaccessibility" to some of his own peculiar "ideas," he never treated her as he did Camden Town when he charged it with "Philistinism" for refusing to be converted to the Arnold faith. If the author of *Literature and Dogma* had been a doctor instead of a poet and a critic, he would hardly have felt it possible to acquit his darling Oxford of "Philistinism," defined as "Inaccessibility to Ideas," with a good conscience. Notwithstanding the presence of Dr. Burdon Sanderson, and one or two other famous illuminati, Oxford seems to be as sublimely indifferent to the "ideas" of modern physiology as she is to German literature or to Protestant dissent.

The local theatre, thinks a correspondent of the *Oxford Chronicle*, is a place where one may be nightly asphyxiated without extra charge. Whereupon another correspondent, who signs himself "Philoxygen," writes in the following strain to the editor of that newspaper: "The public owe a debt of gratitude to the correspondent whose lively and amusing letter on the asphyxiating atmosphere of the Oxford Theatre appeared in your last issue. To its truth we can all bear witness. But, I would ask, is the theatre the only ill-ventilated building in which people gather together? What about the churches? An article in The Hospital has lately drawn pointed attention to this subject. The atmosphere of many churches is as foul and tainted as that of the theatre, and the provisions for ventilation are quite as imperfect. Where are the doctors? Surely, out of the 38 medical practitioners in Oxford, we may suppose that some at least attend places of worship; but how many of them take any steps to avert the notorious dangers of over-heating and insufficient ventilation? I have a country church 'in my mind's eye' which is regularly frequented by three or four of the resident doctors, and at which the air is simply pestilential at times, causing severe headache, sore throat, and bronchial irritation. Is there no remedy for this state of things? If architects, doctors, clergy, churchwardens are all equally indifferent, why should not the laity bestir themselves? Why not have a Society for the Encouragement of Ventilation, whose members resolve (1) to do all that they can to further the love of pure air, (2) not to attend, so far as possible, any meeting or gathering which is held in ill-ventilated or non-ventilated buildings?"

This newspaper correspondent is evidently a man of "light and leading." He does not believe that the facts of science were invented for the mere purpose of laboratory investigation and learned discussion. If to the statements that some of the churches in Oxford are ill-ventilated and insanitary, and that the theatre is a place where asphyxiation is alarmingly possible, he had added the further averment that the Infirmary, which ought to be the very centre of all that is modern and perfect in physical science, is insanitary in character and obsolete in form, he would have presented an indictment which, in spite of whatever might have been said to the contrary, would have ranked the leaders of social and academic life at Oxford, hopelessly amongst Arnold's "Philistines." There is no escape from this position. Physiological ideas are in the air, and the importance of those ideas is by no means confined to medicine. Mental philosophy is profoundly affected by them, and not only so, but jurisprudence is already feeling their influence, and must constantly feel that influence more and more. To be ignorant of physiology, and of physiology in a practical sense, is to be outside the range of one of the most potent tendencies of the times. For practical purposes, the leaders of the University and of social life, and the doctors, as well as the lawyers and parsons, seem to be quite unconscious that anything is going wrong at Oxford, even though half the young men who attend its churches and other public places of resort are compelled to pass their time in an atmosphere of poison.

Such a separation of science from practice as is here displayed shows intellectual capacity of the most peculiar kind. We do not expect every wold-village and every microscopic conventicle to lay hold of the current knowledge of the times and to utilise it in every available way; but if practical science is to make progress anywhere, it should surely make progress at Oxford. No doubt the University has taken the line of insisting upon a classical rather than a scientific education for its members, and there is really no very great objection to such a course. But though that is so, we cannot suppose that the heads of colleges and the parsons and doctors have a positive dislike for oxygen, and a belligerent preference for vile odours. The thing is inconceivable. Even Newman, with all his astonishing regard for everything that was musty and old, could not, one would think, have been got to affirm a regard for "ancient and fish-like smells."

It is disappointing, to say the least, to find Oxford, Professor Burdon Sanderson notwithstanding, a place of gross physiological darkness. One cannot help a kind of desire to find out the reason. The learned Professor does not surely entertain the same objections to Priestly and Oxygen as he expresses towards Metschnikoff and Phagocytes! Is it that he considers elementary science only fit for the elementary minds of the young men who ought to, but perhaps do not, crowd his physiological laboratory? If that be so, we cannot agree with him. Science will never make its way without practice; and even the young men who study physiology for medical purposes will attach little importance to academical prelections if they find one thing preached in the lecture-room and another practised in the theatre or at church. But perhaps Professor Sanderson does not go to the theatre, and, possibly, not even to church. In that case it would hardly be fair to make him responsible for the "whited sepulchre" of Oxford insanitation. But now that the general question has been brought forward, even though it has been done by the "mere laity," it is to be hoped that the Professor and his physiological and medical colleagues will do something to bring up the sanitary condition of Oxford to the level of her outward beauty. It is not creditable to educated persons that the elements of science, with which everybody is so familiar in books, even in Board school copybooks, should be practically unknown and unutilised in places where the educated public most do congregate.

